# Swine influenza virus infection dynamics in two pig farms; results of a longitudinal assessment

**DOI:** 10.1186/1297-9716-43-24

**Published:** 2012-03-27

**Authors:** Meritxell Simon-Grifé, Gerard E Martín-Valls, María J Vilar, Núria Busquets, Mercedes Mora-Salvatierra, Theo M Bestebroer, Ron AM Fouchier, Margarita Martín, Enric Mateu, Jordi Casal

**Affiliations:** 1 Centre de Recerca en Sanitat Animal (CReSA), UAB-IRTA, Campus de la Universitat Autònoma de Barcelona, 08193 Bellaterra, Barcelona, Spain; 2 Department of Virology, Erasmus Medical Center, Rotterdam, The Netherlands; 3 Departament de Sanitat i Anatomia animals, Universitat Autònoma de Barcelona (UAB), 08193 Bellaterra, Barcelona, Spain

## Abstract

In order to assess the dynamics of influenza virus infection in pigs, serological and virological follow-ups were conducted in two whole batches of pigs from two different farms (F1 and F2), from 3 weeks of age until market age. Anti-swine influenza virus (SIV) antibodies (measured by ELISA and hemagglutination inhibition) and nasal virus shedding (measured by RRT-PCR and isolation in embryonated chicken eggs and MDCK cells) were carried out periodically. SIV isolates were subtyped and hemagglutinin and neuraminidase genes were partially sequenced and analyzed phylogenetically. In F1, four waves of viral circulation were detected, and globally, 62/121 pigs (51.2%) were positive by RRT-PCR at least once. All F1 isolates corresponded to H1N1 subtype although hemagglutination inhibition results also revealed the presence of antibodies against H3N2. The first viral wave took place in the presence of colostral-derived antibodies. Nine pigs were positive in two non-consecutive sampling weeks, with two of the animals being positive with the same isolate. Phylogenetic analyses showed that different H1N1 variants circulated in that farm. In F2, only one isolate, H1N2, was detected and all infections were concentrated in a very short period of time, as assumed for a classic influenza outbreak. These findings led us to propose that influenza virus infection in pigs might present different patterns, from an epidemic outbreak to an endemic form with different waves of infections with a lower incidence.

## Introduction

Swine influenza (SI) is caused by *Influenzavirus *type A. In pigs, the disease is reported to be very similar to human influenza: high fever (40.5-41.7°C), lethargy, coughing and laboured breathing, anorexia and weight loss [1,2]. Sneezing, conjunctivitis, nasal discharge and abortions may also be observed [2]. SI-associated gross lung lesions observed in pigs are mainly those of a viral pneumonia, and are characterized by a broncho-intersticial pneumonia (BIP) [3].

Pigs can be infected with avian, swine and human influenza A viruses, and for that reason, swine has been classically proposed to be the mixing vessel where reassortant influenza strains can arise [4,5]. Although this "mixing vessel" concept is now narrower than some years ago, the recent emergence of a human pandemic influenza A virus harbouring genes thought to be originally of swine origin stressed again the interest in the epidemiology of influenza in pigs [6].

Traditionally, the entry of a new influenza virus in a herd was considered to cause the appearance of the clinical signs in a high percentage of animals [3]. However, Swine Influenza Virus (SIV) seems to be more widespread in pigs than previously thought [7]. Besides, the fact that the incidence of confirmed clinical outbreaks of influenza in pigs is relatively low suggests that in most cases, infections are of a subclinical nature [8-10]. On the other hand, although the persistence of SIV activity after an acute outbreak has been described [11], and the existence of endemically infected herds has been postulated [3,7], the establishment of endemic infections in swine herds has never been demonstrated. Beyond the picture of a classic epidemic outbreak, there is very little knowledge about the dynamics of SIV within pig farms.

The aim of the present study was to assess the dynamics of influenza virus infection in pig farms, through serological and virological follow-ups of two whole batches of pigs from two commercial farrow-to-finish pig farms.

## Materials and methods

### Ethics statement

This study was carried out in strict accordance with the guidelines of the Good Experimental Practices (GEP) standard adopted by the European Union. All experimental procedures were conducted in accordance with the recommendations approved by the Animal and Human Ethics experimentation Committee (CEEAH) of the Universitat Autònoma de Barcelona, that ensures the protection and welfare of the animals used in research, in agreement with the current European Union Legislation.

### Selection of herds

Selection criteria were: a previous knowledge of the serological status of the farm; absence of SIV vaccination and, the willingness of the owner to cooperate in such a long-term survey. In a previous study conducted between 2008 and 2009 [10], SIV seroprevalence in sows and fattening pigs was assessed in 98 Spanish farms, of which two farrow-to-finish farms located in Catalonia (NE Spain) were selected for this study. Farm 1 (F1) was a 300-sows farrow to finish swine farm located in a high pig density area, while Farm 2 (F2) was a farrow-to-finish operation of 90 sows located in a region of low pig density.

Before the start of the present study, 10 gilts, 20 sows and 20 pigs of each age (3, 6, 9, 12, 15 and 20 weeks) were tested serologically (ELISA, CIVTEST-Suis, *Laboratorios *Hipra SA, Amer, Spain) to re-confirm the SIV status of the two farms.

### Farm facilities and biosecurity practices

#### Farm 1 (F1)

In F1, dry and pregnant sows were housed in stalls. Piglets remained with the sows until the 4^th ^week of age, when they were moved to nursery facilities. In nurseries, pigs were housed in three separated and independent outdoor modules, with no temperature or ventilation control systems. At 10 weeks of age, pigs were transferred into two independent buildings for fatteners. Finally, at 16 weeks of age, pigs were moved to finishing facilities, where they remained until sent to the slaughterhouse at 24 weeks of age. Fattening and finishing facilities had natural ventilation and open separations between pens.

The management practices in this farm included the use of all in/all out (AIAO) production in the nursery, but not in the growing-finishing facilities. The main biosecurity measures included the application of quarantine to the replacement stock, presence of a perimetral fence around the farm and the application of a rodent control. However, it is worth noting that biosecurity measures such as presence of bird-proof nets in windows or a changing room with showers were not present in F1.

#### Farm 2 (F2)

In F2, sows were housed in individual stalls during gestation. Piglets were transferred to nurseries at 4 weeks of age, where they remained until the 11^th ^week of age. Then, pigs were transferred to pens for fatteners where they were housed until sent to the slaughterhouse. In this farm, pigs were sent to the slaughterhouse in two sittings, at 21 weeks of age (18 pigs) and at 22 weeks of age (57 pigs) depending on their weight. Nurseries were equipped with a forced ventilation system, while fattening units had natural ventilation; both facilities had open separations between pens. Animals were managed on an AIAO basis until reaching market weight.

In this farm quarantine practices were not applied before introduction of replacement stock. Biosecurity measures applied in F2 included the presence of a perimeter fence around the farm, as well as a control program for rodents. Most of the biosecurity measures aimed at reducing disease introduction from people, such as presence of changing room with showers or clothes and boots provided by the farm, were not applied in F2. It is important to note, however, that only the owner and the veterinarian had direct contact with pigs from this farm. Bird-proof nets in windows were not present in F2.

It is noteworthy that in both herds, the distribution of pigs in the different pens was at random and, in consequence, pigs from different litters or previous pens could be mixed.

### Sampling and data collection

Every time the farm was visited, pigs were clinically inspected and the distribution of pigs per pen was recorded. Between visits, farmers were asked to record any abnormal event or presence of clinical signs. In F1, the follow-up started in July 2009 and ended in December 2009, while in F2 animals were followed between January 2010 and June 2010.

In each herd, a whole batch of 3-weeks-old piglets (all piglets of that age present at the farm) was selected for the study, and animals were identified (ear-tagged) individually. In total, 121 pigs (11 litters) and 79 pigs (8 litters) were sampled in F1 and F2, respectively. Sera from sows were also collected.

Pigs were followed from 3 weeks of age until sent to the slaughterhouse. Nasal swabs of sterile cotton (ref. 300251, Deltalab, Barcelona, Spain) and serum (jugular venipuncture) were taken periodically. After collection, nasal swabs were placed with vigorous shaking in 1 mL of phosphate-buffered saline plus 10% glycerol and antibiotics (1000 units/mL penicillin and 1000 units/mL of streptomycin) immediately after collection and stored at -80°C until tested.

Initially sampling was planned to be carried out weekly between the 3^rd ^and the 13^th ^week of age, and afterwards, at 14 weeks (only nasal swabs that week), 15, 17, 20 and 24 weeks of age. However, F2 was sampled weekly between the 3^rd ^and 21^st ^or 22^nd ^week of age because of the failure to detect SIV during the first weeks of sampling.

### Serology

Sera were examined initially by a commercial ELISA directed to detect antibodies against type A influenza nucleocapside (ELISA, CIVTEST-Suis, *Laboratorios *Hipra SA, Amer, Spain). Also, presence of anti-influenza antibodies in nasal swab suspensions of 3-week-old piglets was assessed by means of a competition ELISA nucleoprotein (NP) using the (ID Screen^® ^Influenza A Antibody Competition, ID VET, Montpellier, France). In this case, nasal swab suspensions were examined at a 1/2 dilution, and known positive and negative samples were used as test controls [12].

Sera collected from sows and finishers (17, 20 and 24 weeks of age in F1 and 17 and 20 weeks of age in F2) were analyzed by the hemagglutination inhibition (HI) assay performed according to standard procedures [2] with 4 hemagglutinin units (HU) per well. Cut-off of HI was set to ≥ 1:20 as reported before [9,13]. Three SIV strains that belonged to Eurasian clusters circulating in Europe were used for HI: A/swine/Neth/Best/96 (avian-like H1N1), A/swine/Gent/7625/99 (triple reassortant H1N2), A/swine/Neth/St Oedenrode/96 (avian-like H3N2) (all of them provided by GD, Animal Service Center, Deventer, The Netherlands). Viral stocks were produced in MDCK cells and a single viral batch was used for all HI tests. For those pigs found to be viral shedders more than once, sera were also examined in HI using the isolate previously retrieved from those pigs.

### Nasal shedding of SIV

Detection of SIV in nasal swabs was assessed by means of a Taq-Man real time reverse transcriptase/polymerase chain reaction (RRT-PCR) directed to the detection of the *M *gene of influenza A viruses [12] performed in a Fast7500 equipment (Applied Biosystems, Foster City, CA, USA). Viral RNA was extracted with QIAamp viral kit (Qiagen, Valencia, CA, USA) according to the instructions of the manufacturer.

All SIV RRT-PCR positive samples were inoculated into specific pathogen free (SPF) embryonated chicken eggs (ECE) in order to attempt SIV isolation [2]. Briefly, nasal swab suspensions were centrifuged, and 100 μL of the supernatant were inoculated into the allantoic cavity of 9-11-day-old ECE. Allantoic fluid was harvested 3 days after inoculation, and SIV presence was detected by hemagglutination. Non hemagglutinating allantoic fluids in the first passage were inoculated again in ECE. If the allantoic fluid was negative after the second passage, nasal swab suspensions were inoculated in Madin-Darby Canine Kidney (MDCK) cells cultured with added trypsin (5 μg/mL). Cell culture supernatants were collected at approximately 75% of cytopathic effect, centrifuged and later tested by RRTPCR. Samples that did not produce cytophatic effect were subjected to a second passage in MDCK cells. Samples were discarded if negative after the second passage.

### Subtyping and phylogenetic analysis

Viral isolates were subtyped by multiplex RT-PCR described by Chiapponi et al. [14] for the detection of H1, H3, N1 and N2 genes and sequenced using Big Dye Terminator v3.1 cycle sequencing Kit (Applied Biosystems, Madrid, Spain) and the ABI Prism 3100 sequence analyser (PerkinElmer, Madrid, Spain). The isolates that could not be amplified and sequenced using the methodology cited were analyzed with different primers as an alternative to subtype these strains. Moreover, these primers were also used to sequencing a long fragment of the HA (1676 bp) and NA (1349 bp) genes of 26 isolates randomly selected from the isolates obtained in the different weeks of sampling. Finally, the internal genes from one isolate of 3, 7 and 13 weeks of age were partially sequenced. The sequences of the primer set used to amplify each segment are shown in Additional file 1: Table S1.

Comparison with published sequences (available at NCBI) was carried out using CLUSTAL W, and the unrooted phylogenetic trees were generated by the distance-based neighbor-joining method (1000 iterations) using MEGA 4.1. Relevant and not redundant HA and NA sequences from different countries, species and years were included in the phylogenetic analysis. GenBank accession numbers for all sequences used in this study are listed in Additional file 2: Table S2.

### Statistical analysis

SPSS 15.0 (SPSS Inc., Chicago, IL, USA) was used for all statistical analysis.

## Results

### Farm 1

#### Antibodies against influenza A viruses

**Figure 1:**
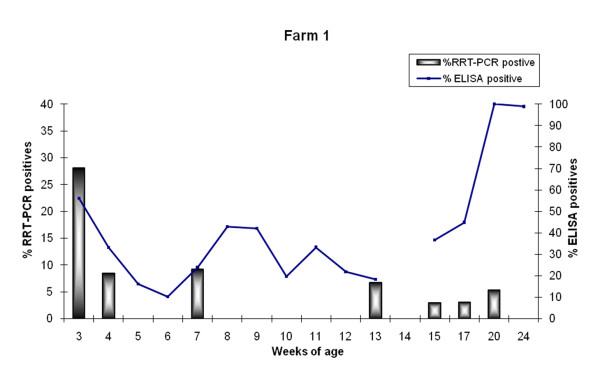
**Seroprevalence and incidence of SIV in Farm 1**. Antibodies against SIV were analyzed by ELISA (line) and nasal shedders were determined by RRT-PCR (bars) at each sampling time.

Figure 1 summarizes the evolution of seroprevalence. At 3 weeks of age, seroprevalence by ELISA was 56.2% (68/121), and declined to a 10.3% (12/116) at 6 weeks of age. Afterwards and until the 15^th ^week of age, seroprevalence varied between 18.3 and 44.9%. Almost all the 15-week-old seronegative animals seroconverted afterwards. Anti-NP antibodies were not detected in nasal swabs from animals of 3 weeks of age.

Table 1 shows the proportion of pigs positive by the hemagglutination inhibition test (≥ 1:20) to H1N1, H1N2 and H3N2 subtypes at 17, 20 and 24 weeks of age. In all cases H1N1 and H3N2 seropositive animals were detected, but no antibodies against H1N2 were found. When the sera from pigs that were positive by RRT-PCR more than once were analyzed by HI test using the strain previously isolated from them as antigen, only 4/9 showed titres ≥ 1:20 at the time of the second detection. These sera with antibodies against the strain isolated in the farm belonged to animals of 7 weeks of age (1/9), 13 weeks of age (2/9) and 15 weeks of age (1/9), while sera without antibodies were from pigs of 7 weeks of age (2/9), 13 weeks of age (2/9) and 24 weeks of age (1/9).

**Table 1:** Seroprevalence of antibodies against H1N1, H1N2 and H3N2 in Farm 1 obtained by HI test

Percentage of seropositive animals*						
Age (weeks)	H1N1		H1N2		H3N2	
	**Seroprevalence (%)**	**95% CI**	**Seroprevalence (%)**	**95% CI**	**Seroprevalence (%)**	**95% CI**

17	83.7	74.5-90.1	0	0-4.7	80.6	71.1-87.6
20	51.6	41.2-61.9	0	0-4.8	96.8	90.5-99.1
24	53.2	42.7-63.5	0	0-4.9	77.7	67.7-85.3

* Cut-off = ≥ 1:20

CI, exact binomial confidence interval

Regarding sows, all were seropositive for H3N2 and 9/11 had antibodies against H1N1.

#### Viral shedding

Using RRT-PCR, 62 animals (51.2%) were positive at least once. As shown in Figure 1, four waves of viral circulation were observed: in farrowing units (at 3 and 4 weeks of age), in nurseries (at 7 weeks of age), in fattening units (at 13 weeks of age), and in finishing units (at 15, 17, and 20 weeks of age), with incidences ranging from 3.0 to 28.1%. Interestingly, nine animals (7.4%) were positive in at least two different occasions.

S IV was isolated either in ECE or MDCK in at least one sample from all weeks, in exception of the 17 weeks of age, that had RRT-PCR positive nasal swabs; namely 42 isolates (58.3% of the positive samples). Isolations were obtained from animals with ages of 3 weeks (19 isolates), 4 (8 isolates), 7 (8 isolates), 13 (4 isolates), 15 (2 isolates), and 20 (1 isolates). Of the 42 isolates, 40 were partially sequenced for hemagglutinin (HA), 34 for neuraminidase (NA) and 34 were subtyped for both HA and NA. Two isolates could not be amplified and sequenced neither HA nor NA.

The similarity of the complete nucleotide sequences of the HA (1676 bp) and NA (1349 bp) from the 26 isolates analyzed ranged from 99.2% to 100% and from 99.4% to 100% for HA and NA genes, respectively. On the other hand, analysis of the nucleotide sequences of the internal genes of three isolates showed a high similarity; from 99.7% to 99.8% for polymerase gene 2 (PB2) and polymerase gene 1 (PB1), from 99.6 to 99.9 for polymerase gene A (PA), from 99.8 to 100% for nucleoprotein gene (NP), of 100% for matrix gene (MA) and from 99.2 to 99.6 for non-structural gene (NS).

**Figure 2:**
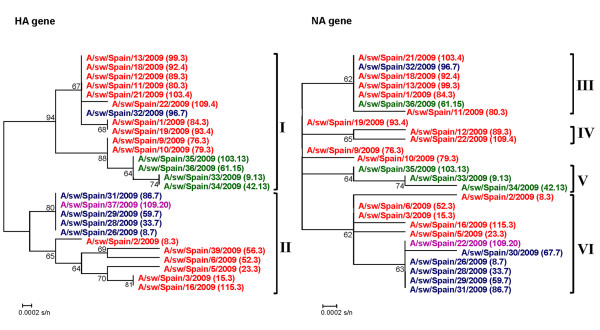
**Phylogenetic tree of the HA1 and NA1 genes of SIV isolates from Farm 1**. The accession numbers of sequence data of influenza virus were deposited in GenBank under the accession numbers [GenBank: JF960169, JF960172 - JF960174, JF960176, JF960177, JF960180 - JF960184, JF960187, JF960189, JF960190, JF960192, JF960193, JF960197, JF960199 - JF960208, JQ301920 - JQ301944]. The strains are indicated by the isolate name and between brackets by the animal number following by the age of animals in which the virus was isolated (in weeks). Strains given in red correspond to available isolates from pigs of 3 and 4 weeks of age. Strains given in blue correspond to available isolates from pigs of 7 weeks of age. Strains given in green correspond to available isolates from pigs of 13 and 15 weeks of age. Strains given in purple correspond to available isolates from pigs of 20 weeks of age. Abbreviations: cluster I, I; cluster II, II; cluster III, III; cluster IV, IV; cluster V, V; and cluster VI, VI.

The phylogenetic analysis of the HA gene showed two distinct clusters designated as I and II (Figure 2). Cluster I was made up of isolates belonging mainly to farrowing area (3 and 4 weeks of age) and to fattening area (13 and 15 weeks of age). In contrast, cluster II was composed of isolates belonging to farrowing area (3 and 4 weeks of age), weaning area (7 weeks of age) and finishing area (20 weeks of age). The NA phylogenetic analysis showed at least 4 different clusters designated as III, IV, V, VI (Figure 2). Cluster III and V included isolates belonging mainly to farrowing area and to fattening area. Cluster IV was made up of isolates from pigs of 3 and 4 weeks of age. Finally, cluster VI was composed of isolates belonging to farrowing area, weaning area and finishing area.

Interestingly, nine animals were found to be positive by RRT-PCR at two sampling times. The SIV could be isolated at the two sampling points only from three out of the nine positive animals (designed as 8, 103 and 109). SIV isolated from the animals 8 and 103 were grouped in cluster II and in cluster I, respectively. In contrast, the distinct isolates obtained from pig 109 were classified in cluster I (isolate obtained at 4 weeks of age) and in cluster II (isolate obtained at 20 weeks of age).

**Figure 3:**
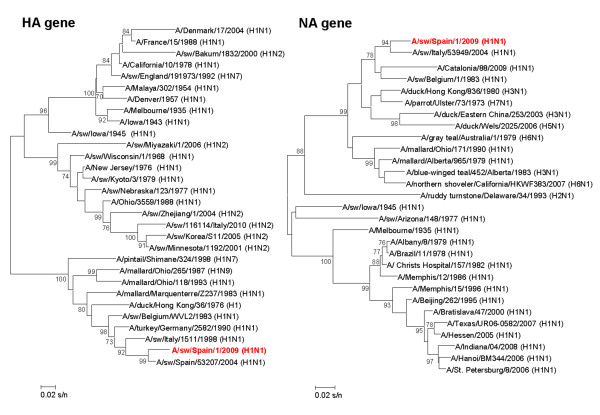
**Phylogenetic analysis for HA and NA of SIV isolates retrieved in Farm 1**. The strains isolated in our study are highlighted in red. Unrooted bootstrapped neighbour-joining trees of nucleotide sequences of hemagglutinin and neuraminidase. Bootstrap values, calculated on 1000 replicated trees, are shown if ≥ 70 percent. Scale bars indicate substitutions per site. The accession numbers of sequence data are provided in Additional file 2: Table S2.

All the isolates from this farm were subtyped as H1N1 and grouped with other European H1N1 SIV of an avian-like clade (Figure 3).

**Figure 4:**
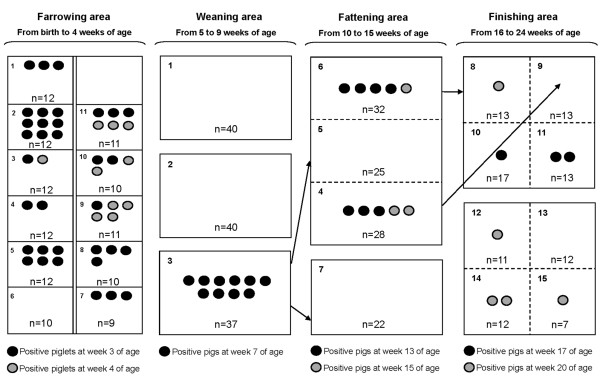
**Distribution of viral shedders according to RRT-PCR results in Farm 1**. Open separations between pens are represented as dashed lines; closed separations between pens are shown with solid lines. Arrows show movements of RRT-PCR positive pigs.

When the distribution of RRT-PCR positive pigs was examined, it was shown that in farrowing units 10/11 litters had at least one positive piglet at 3 weeks of age, but this proportion decreased to 4/11 one week later. In nurseries, all positive pigs of 7 weeks of age were housed in the same pen. In the other two pens viral shedders were not found throughout the whole 6 week period for which they were allocated there. Virus positive animals at 13^th ^and 15^th ^weeks of age were detected in two pens (4 and 6). Finally, for finishers 6/8 pens had at least one positive animal at 17 or 20 weeks of age. The distribution of positive animals throughout the study is represented in Figure 4.

#### Clinical signs and gross lesions

Only a low percentage of pigs (≤ 4%) showed mild influenza-like signs throughout the study, but mortality rates reached 20.3%. When possible, the necropsy of these animals was performed but only two of the nine necropsied pigs presented lesions compatible with BIP. Besides this, two pigs had fibrous/fibrinous polyserositis, and another pig had pulmonary haemorrhage and necrosis compatible with *Actinobacillus pleuropneumoniae*. Taken together, the lesions observed seem to indicate that this farm was affected by a porcine respiratory disease complex.

No viral RNA was detected by RRT-PCR in the lungs of any of the necropsied pigs.

### Farm 2

#### Antibodies against influenza A viruses

**Figure 5:**
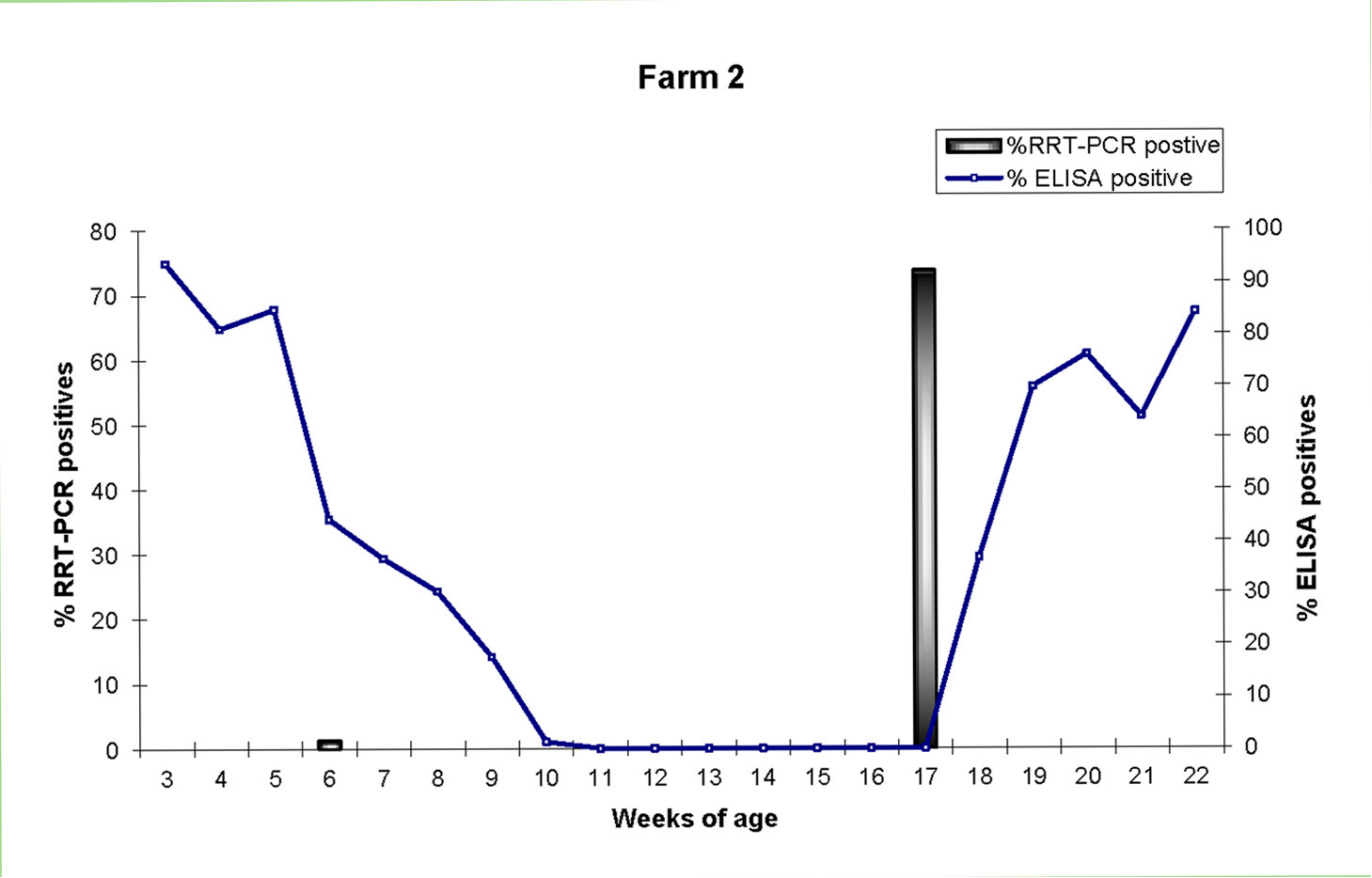
**Seroprevalence and incidence of SIV in Farm 2**. The techniques and symbols used are the same as those used in Figure 1.

In the first sampling (3 weeks of age) seroprevalence by ELISA was 93.7% (74/79). Then, seroprevalence decreased, and by 11 weeks of age all pigs were seronegative and remained so until 17 weeks of age. Seroconversions started at 18 weeks of age and in the last sampling (22 weeks of age) 84.2% (48/57) of animals were seropositive. Figure 5 summarizes these results. Anti-NP antibodies were not detected in nasal swabs from animals of 3 weeks of age.

Using HI, at 20 weeks of age, 92% (69/75) of the pigs were seropositive against H1N2, but no antibodies against H1N1 or H3N2 were found. Regarding sows, 4/8 were positive for H1N2 and 7/8 and 8/8 were seropositive to H1N1 and H3N2, respectively.

#### Viral shedding

Fifty-seven pigs (72.2%) were positive by RRT-PCR; of them, one was positive at 6 weeks of age and all the others at 17 weeks of age.

**Figure 6:**
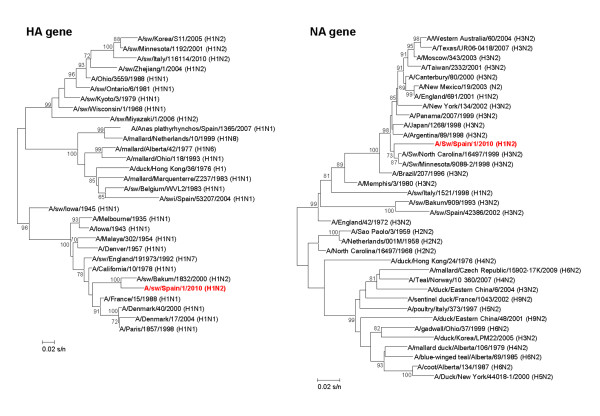
**Phylogenetic analysis for HA and NA of SIV isolates retrieved in Farm 2**. Color scheme, rooting, and scale are the same as those used in Figure 3. The accession numbers of sequence data are provided in Additional file 2: Table S2.

No viral isolation was obtained from nasal swabs in ECE; while, 53 isolates were obtained in MDCK cells, representing 92.9% of RRT-PCR positive samples. Unfortunately, SIV could not be isolated from the only RRT-PCR positive nasal swab at 6 weeks. For further characterization seven isolates were randomly selected, and their HA and NA genes were partially sequenced, corresponding to H1N2 SIV. Analysis of the nucleotide sequences of the HA (382 bp) and NA (702 bp) showed a similarity ranging from 99.4% to 100% and from 99.8 to 100%, for HA and NA genes respectively, indicating the presence of just one viral strain. The H1 sequences were phylogenetically related to one SIV isolated in 2000 in Germany (A/swine/Bakum/1832/2000 (H1N2)), and could be classified in a cluster where human and swine influenza viruses are included. In contrast, the N2 sequences were grouped with North-American SIV corresponding to H3N2 subtype (Figure 6). Positive animals detected in week 17th were distributed among all the pens that housed fatteners.

#### Clinical signs and gross lesions

Mild clinical signs compatible with influenza were detected at 17^th ^and 18^th ^weeks of age, but only affected a low percentage of pigs (6.6% and 1.3%, respectively). The mortality rate in this batch during the study was 5.1%.

## Discussion

Results of the present study illustrate the existence of epidemic and endemic influenza infections in pig farms. While the presentation of the infection in F2 agrees with the traditional picture of an epidemic form of influenza, although of low virulence, the pattern of F1 depicts an endemic situation with an insidious spread, no overt signs but high mortality, and with the co-circulation of different H1N1 variants and probably an additional H3N2, as shown by HI. This opens several questions about the epidemiology, the clinical significance and even about the protection against infection with similar strains of SIV.

In F1 four viral waves were detected followed by seroconversion of a number of pigs. In this farm we identified shedder piglets at 3 and 4 weeks of age, while they were still with the sows. This finding contrasts with previous data suggesting that most SIV infections take place after the decay of maternal antibodies which occurs after 10 weeks of age [15,16]. On the other hand, mucosal IgA is considered to be a correlate of protection against virus replicating in the upper airways [17,18]. In the present case, piglets with maternal-derived antibodies against SIV were found to be positive by RRT-PCR, reinforcing the idea that the measurement of maternal antibodies does not correlate with protection against SIV at a mucosal level [19-23]. All the SIV positive piglets of 3-4 weeks of age showed a lack of specific IgA anti- SIV in nasal level. Beyond a potential lack of sensitivity of the test for the detection of antibodies in nasal mucus, this result would explain the finding of seropositive but infected piglets.

Interestingly, nine pigs were detected as positive by RRT-PCR at two sampling times separated at least four weeks. Although it may be possible that these pigs were infected continuously, but positivity was not detected at some of the sampling times, it seems unlikely since such a long duration of SIV nasal viral shedding has never been reported [24].

Humoral protection against influenza viruses is mainly mediated by antibody responses to HA [25]. In this sense, we also identified two pigs infected in different weeks by SIV strains belonging to the same HA clade in spite of having HI titres > 1:20 against the infecting strain. This observation can be either the result of a true infection, in which case the predictive value of HI antibodies for determining protection could be questioned, or the consequence of an external contamination of the sample, produced, for example by a recent contact of the pig with a shedder pen-mate.

The presence of infected piglets in farrowing units also raises the question of the potential sources of infection. The most obvious source of virus for the piglets could be the sows, although most of them were seropositive for H1N1. Unfortunately we did not test them virologically, and this point cannot be clarified. In any case, the role of sows for maintaining viral circulation in SIV endemic farms is unknown and would deserve more in-depth studies.

One of the most interesting findings of the study was the detection of different H1N1 variants in the same batch of pigs from F1 accordingly the phylogenetic analysis of HA and NA instead the internal genes from the three isolates analyzed showed a high similarity between them. Moreover, the isolates seem to be grouped in the different clusters according to the weeks of age of the animals. Taken together, these results suggest that drift processes have occurred in F1 and as a consequence drift variants have been generated during the sampling frame. To our knowledge this is the first report of some close related H1N1 variants co-circulating endemically in a herd. Besides this, the existence of variants belonging to the H1N1 subtype with small genetic divergence suggests that this virus have been circulating in the herd for a long time. The endemic circulation of distinct H1N1 strains in F1 emphasizes the potential for the emergence of reassortant viruses in pig farms. However, the evidence of simultaneous infection of the same pigs is still lacking. Interestingly, recent studies have shown that intra-subtype reassortment events have played an important role in the evolutionary history of A/H1N1; for example, in the genesis of strains associated with influenza epidemics in humans caused by A/H1N1 viruses in 1947 and 1951 [26]. Furthermore, the presence of drift variants in the same batch of pigs may explain the detection of positive pigs by RRT-PCR more than once sampling time since antigenic drift may facilitate viral escape from antibody neutralization [27]. These facts can be also explained by the development of a weak immunity against the homologous or the heterologous strain, suggesting a partial protection unable to prevent the second infection. However, these finding should be thoroughly investigated by means of transmission-by-contact models.

Another point of interest is the source of SIV infection in the studied farms. The introduction of asymptomatic carrier pigs as well as the transmission from humans could explain the introduction of the virus in these farms. Furthermore, the dissemination of the virus from a neighbouring farm, by aerial transmission could be another potential mechanism of SIV introduction [3]. In this sense, F1 was located in a region of higher pig density areas compared to where F2 was located, and since pig density in a region has been related with SIV prevalence [28] it may seem that F1 was at a higher risk of SIV introduction compared to F2. Finally, other possible means of SIV introduction in these farms could be via fomites or birds.

Influenza viruses are usually classified into Eurasian and north American lineages. The phylogenetic analysis of strains isolated in F2 revealed that the NA was more related to those of swine and human H3N2 virus from North-American lineage. These findings are in agreement with an earlier study by Liu et al. [29], who proposed that the classification of influenza viruses should be more complicated than these two lineages. Moreover, these results highlight the potential intercontinental virus exchange, gene flow and reassortant between Eurasian and North American lineages.

Closed separations between pens which do not allow direct physical contact between pigs from different pens are often considered as a preventive measure against dissemination of airborne pathogens, including SIV [10,30]. In our study, the lack of transmission among pigs housed in different pens with closed separations indicates that it would be advisable to design farm facilities with closed partitions between pens in order to minimize spread of SIV infection.

From a methodological point of view, it is worth to note that only H1N1 and H1N2 viruses could be isolated in spite of evidences for H3N2 being present in F1. Multiples reasons could explain the unsuccessful isolation of this subtype, among them the inactivation of the virus during transport or failure to replicate in eggs [31] or in MDCK cells cultures [32,33] due to a low HA receptor-binding activity. The inactivation hypothesis seems unlikely because of the considerable rate of isolation for other samples treated exactly the same than those containing H3N2. Furthermore, some of the isolates could not be sequenced because RT-PCR failed to amplify the HA or NA genes. In any case, these results can be interpreted as the consequence of a high variability of HA sequences or eventually, can also be attributed to other circulating HA and NA different to H1, H3, N1 or N2. Regarding HI test, it is important to consider that the strains used as antigen were from The Netherlands and Belgium, and all of them were at least 12 years old. The use of these strains can result in an underestimation of the true percentage of seropositive animals. However, in a recent study [34] on cross reactivity between A/swine/Neth/Best/96 and A/swine/Spain/53207/2004, both strains produced fairly similar titres. These data suggest that, at least for H1N1, the results of the present study were not substantially affected by the use of Dutch or Belgian strains. Furthermore, it is important to note that despite the high specificity (100%) of the indirect ELISA, the sensitivity (Se) seems be better for H1N1 subtype than for H1N2 and H3N2 subtypes (Se = 100%, 86.9% and 73.4%, respectively) [35], and this could also result in an underestimation of the seroprevalences obtained.

In conclusion, we report that influenza infection in pigs from commercial herds can occur with different patterns, from an acute outbreak with epidemic spread to an endemic situation. This work also shows that SIV infection can occur in piglets in presence of colostral-derived antibodies against the subtypes circulating in the farm. Moreover, evidences suggest that homologous protection after infection with one strain could not fully prevent a second infection with the same strain or a closely related one. Also, in an endemic farm, several SIV may co-circulate for extended periods of time. A better knowledge of the SIV epidemiology may contribute to improve the understanding of the arising of pandemic viruses.

## Abbreviations

ELISA: Enzyme linked-immunosorbent assay; MDCK: Madin-Darby canine Kidney.

## Competing interests

The authors declare that they have no competing interests.

## Authors' contributions

MSG participated in the design of the study, carried out the sample collection, performed the experiments and the statistical analysis and wrote the paper. GEMV participated in the design of the study, performed the experiments and helped in the sample collection. MJV participated in the sample collection and immunoassays NB participated in molecular studies and helped to draft the manuscript. RAMF participated in molecular studies and helped to draft the manuscript. TMB helped in molecular studies. MMS helped in the sample collection and participated in molecular studies and immunoassays. MM conceived of the study, and participated in its design and coordination and helped to draft the manuscript. EM conceived of the study, and participated in its design and coordination and helped to draft the manuscript. JC conceived of the study, and participated in its design and coordination and helped to draft the manuscript. All authors read and approved the final manuscript.

## Supplementary Material

Additional file 1 **Table S1 Primer set used to amplify each segment of the SIV**. Primer set used to amplify each segment of the SIV and information about the begin and end positions of each one. Click here for file

Additional file 2 **Table S2 GenBank accession numbers of HA and NA sequences used in phylogenetic analysis**. GenBank accession numbers and background information for sequences of influenza A virus used in the phylogenetic analysis. Click here for file
