# Supplementary material for: Swine influenza virus infection dynamics in two pig farms; results of a longitudinal assessment
Source: Vet Res. 2012 Mar 27;43(1):24. doi: 10.1186/1297-9716-43-24 (PMC3353254; doi:10.1186/1297-9716-43-24)
Supplement: Additional file 1 — Table S1 Primer set used to amplify each segment of the SIV. Primer set used to amplify each segment of the SIV and information about the begin and end positions of each one. [file 1297-9716-43-24-S1.DOC]

**Table S1. Primer set used to amplify each segment of the SIV. Primer set used to amplify each segment of the SIV and information about the begin and end positions of each one.**

| Gene | Forward primer (5’-3’) | Reverse primer (5’-3’) |
| --- | --- | --- |
| PB2 | 1-AGCAAAAGCAGGTCAA-16 | 2341-AGTAGAAACAAGGTCGTTTTTAAAC-2316 |
| PB2 | 533-ATGGAAGTTGTTTTCCC-550 | 1622-CTCCCACATCATTGACGATG-1602 |
| PB2 | 974-ATATGCAAGGCTGCAATGGG-994 |  |
| PB2 | 1640-TCATCGTCAATGATGTGGGA-1660 |  |
| PB1 | 1-AGCGAAAGCAGGCAAACCATTTGAATG-27 | 785-CTTTTGTCATTGTGTTCAGTGTCAGTGC-712 |
| PB1 | 598-AGGGACAACATGACCAAGAAAATG-621 | 1092-AGCTTCATGCTCTTACTTTCGAAC-1115 |
| PB1 | 1036-AGCATTGCTCCTATAATGTTCTC-1058 | 1708-GTTTGAATTTGTGTGTCACCTCTGTC-1733 |
| PB1 | 1621-AATATGATAAACAATGACCTTGG-1643 | 2320-AGTAGAAACAAGGCATTTTTTC-2341 |
| PA | 1-AGCGAAAGCAGGTACTGATCCAAAATGG-28 | 653-GTTCCTGTGATTTCAAATCTTTCTTC-628 |
| PA | 466-TTCTCATTCACTGGGGAGGAAATGGC-491 | 1260-GTTGAATTCATTCTGGATCCAGCTTG-1235 |
| PA | 1123-AAGTGGGCACTTGGTGAGAATATGGC-1148 | 1777-GGCAGCGCCTCATTTCCATTCCCC-1754 |
| PA | 1570-GATGTGGTAAACTTTGTGAGTATGG-1594 | 2233-AGTAGAAACAAGGTACTTTTTTGGAC-2208 |
| HA | 1-AGCAAAAGCAGGGG-14 | 1743-AGTAGAAACAAGGGTGTTTT-1724 |
| NP | 1-AGCAAAAGCAGGGT-14 | 1565-AGTAGAAACAAGGGTATTTTTC-1544 |
| NA | 1-AGCAAAAGCAGGAGT-15 | 1467-AGTAGAAACAAGGAGTTTTTT-1447 |
| NA | 680-TGAGAACACAAGAGTCTGAATGTG-700 | 1140-TTCGGATCCCAAATCATCTC-1120 |
| MA | 1-AGCAAAAGCAGGTAGAT-17 | 1027-AGTAGAAACAAGGTAGTTTTTTACTC-1002 |
| NS | 1-AGCAAAAGCAGGGTG-15 | AGTAGAAACAAGGGTGTTTTTTA |

Abbreviations: polymerase genes, PB2, PB1, PA; hemagglutinin gene, HA; nucleoprotein gene, NP; neuraminidase gene, NA; matrix gene, MA; non-structural gene, NS.
