# Supplementary material for: Swine influenza virus infection dynamics in two pig farms; results of a longitudinal assessment
Source: Vet Res. 2012 Mar 27;43(1):24. doi: 10.1186/1297-9716-43-24 (PMC3353254; doi:10.1186/1297-9716-43-24)
Supplement: Additional file 2 — Table S2 GenBank accession numbers of HA and NA sequences used in phylogenetic analysis. GenBank accession numbers and background information for sequences of influenza A virus used in the phylogenetic analysis. [file 1297-9716-43-24-S2.DOC]

**Table S2. GenBank accession numbers of HA and NA sequences used in phylogenetic analysis.** GenBank accession numbers and background information for sequences of influenza A virus used in the phylogenetic analysis.

|  | **GenBank Accession** | **Country of Isolation** | **Year of Isolation** | **Isolate (subtype)** |
| --- | --- | --- | --- | --- |
| 1 | CY009326 | Australia | 1935 | A/Melbourne/1935 (H1N1) |
| 2 | CY009324 | Australia | 1935 | A/Melbourne/1935 (H1N1) |
| 3 | CY020461 | USA | 1943 | A/Iowa/1943 (H1N1) |
| 4 | EU139834 | USA | 1945 | A/swine/Iowa/1945 (H1N1) |
| 5 | EU139824 | USA | 1945 | A/swine/Iowa/1945 (H1N1) |
| 6 | CY021053 | Malaysia | 1954 | A/Malaya/302/1954 (H1N1) |
| 7 | CY008988 | USA | 1957 | A/Denver/1957 (H1N1) |
| 8 | CY077742 | Netherlands | 1958 | A/Netherlands/001M/1958 (H2N2) |
| 9 | AY209901 | Brazil | 1959 | A/Sao Paolo/3/1959 (H2N2) |
| 10 | EU139825 | USA | 1968 | A/swine/Wisconsin/1/1968 (H1N1) |
| 11 | CY031589 | USA | 1968 | A/North Carolina/1/1968 (H2N2) |
| 12 | AY210132 | UK | 1972 | A/England/42/1972 (H3N2) |
| 13 | K02252 | Ireland | 1973 | A/parrot/Ulster/1973 (H7N1) |
| 14 | CY021957 | USA | 1976 | A/New Jersey/1976 (H1N1) |
| 15 | D00838 | Japan | 1976 | A/duck/Hong Kong/36/1976 (H1) |
| 16 | D21184 | Japan | 1976 | A/duck/Hong Kong/24/1976 (H4N2) |
| 17 | CY022373 | USA | 1977 | A/swine/Nebraska/123/1977 (H1N1) |
| 18 | CY025004 | USA | 1977 | A/swine/Arizona/148/1977 (H1N1) |
| 19 | CY004458 | USA | 1977 | A/mallard/Alberta/42/1977 (H1N6) |
| 20 | CY021717 | USA | 1978 | A/California/10/1978 (H1N1) |
| 21 | CY020295 | Brazil | 1978 | A/Brazil/11/1978 (H1N1) |
| 22 | CY014624 | Australia | 1979 | A/gray teal/Australia/1/1979 (H6N1) |
| 23 | CY004492 | USA | 1979 | A/mallard/Alberta/965/1979 (H1N1) |
| 24 | CY026413 | Albany | 1979 | A/Albany/8/1979 (H1N1) |
| 25 | AB434384 | Japan | 1979 | A/swine/Kyoto/3/1979 (H1N1) |
| 26 | CY004792 | USA | 1979 | A/mallard duck/Alberta/106/1979 (H4N2) |
| 27 | AB292403 | Japan | 1980 | A/duck/Hong Kong/836/1980 (H3N1) |
| 28 | CY008470 | USA | 1980 | A/Memphis/3/1980 (H3N2) |
| 29 | CY026451 | Canada | 1981 | A/swine/Ontario/6/1981 (H1N1) |
| 30 | CY021039 | UK | 1982 | A/Christs Hospital/157/1982 (H1N1) |
| 31 | CY004636 | USA | 1983 | A/blue-winged teal/Alberta/452/1983 (H3N1) |
| 32 | AJ412690 | Belgium | 1983 | A/swine/Belgium/1/1983 (H1N1) |
| 33 | GU066779 | France | 1983 | A/mallard/Marquenterre/Z237/2007 (H1N1) |
| 34 | CY037967 | Belgium | 1983 | A/swine/Belgium/WVL2/1983 (H1N1) |
| 35 | CY004196 | USA | 1985 | A/blue-winged teal/Alberta/69/1985 (H6N2) |
| 36 | CY019103 | USA | 1986 | A/Memphis/12/1986 (H1N1) |
| 37 | CY017275 | USA | 1987 | A/mallard/Ohio/265/1987 (H1N9) |
| 38 | CY004517 | Alberta | 1987 | A/coot/Alberta/134/1987 (H6N2) |
| 39 | CY024925 | USA | 1988 | A/Ohio/3559/1988 (H1N1) |
| 40 | L19019 | France | 1988 | A/France/15/1988 (H1N1) |
| 41 | U96766 | Germany | 1990 | A/turkey/Germany/2482/1190 (H1N1) |
| 42 | CY012802 | USA | 1990 | A/mallard/Ohio/171/1990 (H1N1) |
| 43 | U85986 | UK | 1992 | A/swine/England/191973/1992 (H1N7) |
| 44 | CY015137 | USA | 1993 | A/ruddy turnstone/Delaware/81 1993 (H2N1) |
| 45 | CY018885 | USA | 1993 | A/mallard/Ohio/118/1993 (H1N1) |
| 46 | EF409255 | Germany | 1993 | A/swine/Bakum/909/1993 (H3N2) |
| 47 | CY033616 | China | 1995 | A/Beijing/262/1995 (H1N1) |
| 48 | CY021007 | USA | 1996 | A/Memphis/15/1996 (H1N1) |
| 49 | EF584357 | Brazil | 1996 | A/Brazil/207/1996 (H3N2) |
| 50 | CY020231 | Italy | 1997 | A/poultry/Italy/373/1997 (H5N2) |
| 51 | AB274304 | Japan | 1998 | A/pintail/Shimane/324/1998 (H1N7) |
| 52 | AJ344016 | Italy | 1998 | A/swine/Italy/1511/1998 (H1N1) |
| 53 | AJ457892 | France | 1998 | A/Paris/1857/1998 (H1N1) |
| 54 | AF153238 | USA | 1998 | A/Swine/Minnesota/9088-2/1998 (H3N2) |
| 55 | EF584359 | Japan | 1998 | A/Japan/1268/1998 (H3N2) |
| 56 | EF584374 | Argentina | 1998 | A/Argentina/89/1998 (H3N2) |
| 57 | AJ412701 | Italy | 1998 | A/swine/Italy/1521/1998 (H1N2) |
| 58 | CY060206 | Netherlands | 1999 | A/mallard/Netherlands/10/1999 (H1N8) |
| 59 | AF268139 | USA | 1999 | A/Swine/North Carolina/16497/1999 (H3N2) |
| 60 | CY016158 | USA | 1999 | A/gadwall/Ohio/37/1999 (H6N2) |
| 61 | DQ487337 | Panama | 1999 | A/Panama/2007/1999 (H3N2) |
| 62 | EU053148 | Germany | 2000 | A/swine/Bakum/1832/2000 (H1N2) |
| 63 | AJ518093 | Slovakia | 2000 | A/Bratislava/47/2000 (H1N1) |
| 64 | EU097942 | Denmark | 2000 | A/Denmark/40/2000 (H1N1) |
| 65 | AY300933 | USA | 2000 | A/Duck/New York/44018-1/2000 (H5N2) |
| 66 | CY008782 | UK | 2000 | A/Canterbury/80/2000 (H3N2) |
| 67 | EU139828 | USA | 2001 | A/swine/Minnesota/1192/2001 (H1N2) |
| 68 | EU429707 | China | 2001 | A/duck/Eastern China/48/2001 (H9N2) |
| 69 | DQ249253 | Taiwan | 2001 | A/Taiwan/2332/2001 (H3N2) |
| 70 | AJ489847 | UK | 2001 | A/England/691/2001 (H1N2) |
| 71 | AM157359 | France | 2002 | A/sentinel duck/France/1043/2002 (H9N2) |
| 72 | CY000443 | USA | 2002 | A/New York/134/2002 (H3N2) |
| 73 | CY020503 | Spain | 2002 | A/swine/Spain/42386/2002 (H3N2) |
| 74 | EU429754 | China | 2003 | A/duck/Eastern China/253/2003 (H3N1) |
| 75 | EU100577 | Mexico | 2003 | A/New Mexico/19/2003 (N2) |
| 76 | DQ091199 | Russia | 2003 | A/Moscow/343/2003 (H3N2) |
| 77 | EU045388 | Italy | 2004 | A/swine/Italy/53949/2004 (H1N1) |
| 78 | DQ139320 | China | 2004 | A/swine/Zhejiang/1/2004 (H1N2) |
| 79 | EU097943 | Denmark | 2004 | A/Denmark/17/2004 (H1N1) |
| 80 | CY010580 | Spain | 2004 | A/swine/Spain/53207/2004 (H1N1) |
| 81 | EU429745 | China | 2004 | A/duck/Eastern China/6/2004 (H3N2) |
| 82 | CY015950 | Australia | 2004 | A/Western Australia/60/2004 (H3N2) |
| 83 | FJ231817 | Germany | 2005 | A/Hessen/4/2005 (H1N1) |
| 84 | DQ666933 | Korea | 2005 | A/swine/Korea/S11/2005 (H1N2) |
| 85 | EU301289 | Korea | 2005 | A/duck/Korea/LPM22/2005 (H3N2) |
| 86 | AB286007 | Vietnam | 2006 | A/Hanoi/BM344/2006 (H1N1) |
| 87 | CY035128 | Russia | 2006 | A/St. Petersburg/8/2006 (H1N1) |
| 88 | GQ355843 | Austria | 2006 | A/duck/Wels/2025/2006 (H5N1) |
| 89 | AB441170 | Japan | 2006 | A/swine/Miyazaki/1/2006 (H1N2) |
| 90 | CY032714 | USA | 2007 | A/northern shoveler/California/2007 (H6N1) |
| 91 | CY026717 | USA | 2007 | A/Texas/UR06-0582/2007 (H1N1) |
| 92 | FN386463 | Spain | 2007 | A/Anas plathyrhynchos/Spain/ 1365/2007 (H1N1) |
| 93 | CY025853 | USA | 2007 | A/Texas/UR06-0418/2007 (H3N2) |
| 94 | FN773069 | Norway | 2007 | A/Teal/ A/Teal/Norway/10 1360/2007 (H4N2) |
| 95 | EU779649 | USA | 2008 | A/Indiana/04/2008 (H1N1) |
| 96 | GQ166189 | Spain | 2009 | A/Catalonia/88/2009 (H1N1) |
| 97 | HQ244432 | Czech Republic | 2009 | A/mallard/Czech Republic/15902-17K/2009 (H6N2) |
| 98 | JF960169 | Spain | 2009 | A/swine/Spain/1/2009 (H1N1) |
| 99 | JF960172 | Spain | 2009 | A/swine/Spain/1/2009 (H1N1) |
| 100 | JF960173 | Spain | 2009 | A/swine/Spain/2/2009 (H1N1) |
| 101 | JQ301920 | Spain | 2009 | A/swine/Spain/2/2009 (H1N1) |
| 102 | JF960174 | Spain | 2009 | A/swine/Spain/3/2009 (H1N1) |
| 103 | JQ301921 | Spain | 2009 | A/swine/Spain/3/2009 (H1N1) |
| 104 | JF960175 | Spain | 2009 | A/swine/Spain/4/2009 (H1N1) |
| 105 | JQ301945 | Spain | 2009 | A/swine/Spain/4/2009 (H1N1) |
| 106 | JF960176 | Spain | 2009 | A/swine/Spain/5/2009 (H1N1) |
| 107 | JQ301922 | Spain | 2009 | A/swine/Spain/5/2009 (H1N1) |
| 108 | JF960177 | Spain | 2009 | A/swine/Spain/6/2009 (H1N1) |
| 109 | JQ301923 | Spain | 2009 | A/swine/Spain/6/2009 (H1N1) |
| 110 | JF960178 | Spain | 2009 | A/swine/Spain/7/2009 (H1N1) |
| 111 | JQ301946 | Spain | 2009 | A/swine/Spain/7/2009 (H1N1) |
| 112 | JF960179 | Spain | 2009 | A/swine/Spain/8/2009 (H1N1) |
| 113 | JQ301947 | Spain | 2009 | A/swine/Spain/8/2009 (H1N1) |
| 114 | JF960180 | Spain | 2009 | A/swine/Spain/9/2009 (H1N1) |
| 115 | JQ301924 | Spain | 2009 | A/swine/Spain/9/2009 (H1N1) |
| 116 | JF960181 | Spain | 2009 | A/swine/Spain/10/2009 (H1N1) |
| 117 | JQ301925 | Spain | 2009 | A/swine/Spain/10/2009 (H1N1) |
| 118 | JF960182 | Spain | 2009 | A/swine/Spain/11/2009 (H1N1) |
| 119 | JQ301926 | Spain | 2009 | A/swine/Spain/11/2009 (H1N1) |
| 120 | JF960183 | Spain | 2009 | A/swine/Spain/12/2009 (H1N1) |
| 121 | JQ301927 | Spain | 2009 | A/swine/Spain/12/2009 (H1N1) |
| 123 | JF960184 | Spain | 2009 | A/swine/Spain/13/2009 (H1N1) |
| 124 | JQ301928 | Spain | 2009 | A/swine/Spain/13/2009 (H1N1) |
| 125 | JQ301959 | Spain | 2009 | A/swine/Spain/13/2009 (H1N1) |
| 128 | JQ301963 | Spain | 2009 | A/swine/Spain/13/2009 (H1N1) |
| 129 | JQ301966 | Spain | 2009 | A/swine/Spain/13/2009 (H1N1) |
| 130 | JQ301966 | Spain | 2009 | A/swine/Spain/13/2009 (H1N1) |
| 131 | JQ301972 | Spain | 2009 | A/swine/Spain/13/2009 (H1N1) |
| 132 | JQ301975 | Spain | 2009 | A/swine/Spain/13/2009 (H1N1) |
| 133 | JQ301948 | Spain | 2009 | A/swine/Spain/14/2009 (H1) |
| 134 | JQ301949 | Spain | 2009 | A/swine/Spain/15/2009 (H1) |
| 135 | JF960187 | Spain | 2009 | A/swine/Spain/16/2009 (H1N1) |
| 136 | JQ301929 | Spain | 2009 | A/swine/Spain/16/2009 (H1N1) |
| 137 | JF960188 | Spain | 2009 | A/swine/Spain/17/2009 (H1N1) |
| 138 | JQ301950 | Spain | 2009 | A/swine/Spain/17/2009 (H1N1) |
| 139 | JF960189 | Spain | 2009 | A/swine/Spain/18/2009 (H1N1) |
| 140 | JQ301930 | Spain | 2009 | A/swine/Spain/18/2009 (H1N1) |
| 141 | JF960190 | Spain | 2009 | A/swine/Spain/19/2009 (H1N1) |
| 143 | JQ301931 | Spain | 2009 | A/swine/Spain/19/2009 (H1N1) |
| 144 | JF960191 | Spain | 2009 | A/swine/Spain/20/2009 (H1N1) |
| 145 | JQ301951 | Spain | 2009 | A/swine/Spain/20/2009 (H1N1) |
| 147 | JF960192 | Spain | 2009 | A/swine/Spain/21/2009 (H1N1) |
| 148 | JQ301932 | Spain | 2009 | A/swine/Spain/21/2009 (H1N1) |
| 149 | JF960193 | Spain | 2009 | A/swine/Spain/22/2009 (H1N1) |
| 150 | JQ301933 | Spain | 2009 | A/swine/Spain/22/2009 (H1N1) |
| 151 | JQ301952 | Spain | 2009 | A/swine/Spain/23/2009 (H1) |
| 152 | JF960195 | Spain | 2009 | A/swine/Spain/24/2009 (H1N1) |
| 153 | JQ301953 | Spain | 2009 | A/swine/Spain/24/2009 (H1N1) |
| 154 | JQ301954 | Spain | 2009 | A/swine/Spain/25/2009 (H1) |
| 155 | JF960197 | Spain | 2009 | A/swine/Spain/26/2009 (H1N1) |
| 156 | JQ301934 | Spain | 2009 | A/swine/Spain/26/2009 (H1N1) |
| 157 | JF960198 | Spain | 2009 | A/swine/Spain/27/2009 (H1N1) |
| 158 | JQ301955 | Spain | 2009 | A/swine/Spain/27/2009 (H1N1) |
| 159 | JF960199 | Spain | 2009 | A/swine/Spain/28/2009 (H1N1) |
| 160 | JQ301935 | Spain | 2009 | A/swine/Spain/28/2009 (H1N1) |
| 161 | JF960200 | Spain | 2009 | A/swine/Spain/29/2009 (H1N1) |
| 162 | JQ301936 | Spain | 2009 | A/swine/Spain/29/2009 (H1N1) |
| 163 | JF960201 | Spain | 2009 | A/swine/Spain/30/2009 (H1N1) |
| 164 | JQ301956 | Spain | 2009 | A/swine/Spain/30/2009 (H1N1) |
| 165 | JF960202 | Spain | 2009 | A/swine/Spain/31/2009 (H1N1) |
| 166 | JQ301937 | Spain | 2009 | A/swine/Spain/31/2009 (H1N1) |
| 167 | JQ301960 | Spain | 2009 | A/swine/Spain/31/2009 (H1N1) |
| 168 | JQ301964 | Spain | 2009 | A/swine/Spain/31/2009 (H1N1) |
| 169 | JQ301967 | Spain | 2009 | A/swine/Spain/31/2009 (H1N1) |
| 170 | JQ301970 | Spain | 2009 | A/swine/Spain/31/2009 (H1N1) |
| 171 | JQ301973 | Spain | 2009 | A/swine/Spain/31/2009 (H1N1) |
| 172 | JQ301976 | Spain | 2009 | A/swine/Spain/31/2009 (H1N1) |
| 173 | JF960203 | Spain | 2009 | A/swine/Spain/32/2009 (H1N1) |
| 174 | JQ301938 | Spain | 2009 | A/swine/Spain/32/2009 (H1N1) |
| 175 | JF960204 | Spain | 2009 | A/swine/Spain/33/2009 (H1N1) |
| 176 | JQ301939 | Spain | 2009 | A/swine/Spain/33/2009 (H1N1) |
| 177 | JF960205 | Spain | 2009 | A/swine/Spain/34/2009 (H1N1) |
| 178 | JQ301940 | Spain | 2009 | A/swine/Spain/34/2009 (H1N1) |
| 179 | JQ301961 | Spain | 2009 | A/swine/Spain/34/2009 (H1N1) |
| 180 | JQ301965 | Spain | 2009 | A/swine/Spain/34/2009 (H1N1) |
| 181 | JQ301968 | Spain | 2009 | A/swine/Spain/34/2009 (H1N1) |
| 182 | JQ301971 | Spain | 2009 | A/swine/Spain/34/2009 (H1N1) |
| 183 | JQ301974 | Spain | 2009 | A/swine/Spain/34/2009 (H1N1) |
| 184 | JQ301977 | Spain | 2009 | A/swine/Spain/34/2009 (H1N1) |
| 185 | JF960206 | Spain | 2009 | A/swine/Spain/35/2009 (H1N1) |
| 186 | JQ301941 | Spain | 2009 | A/swine/Spain/35/2009 (H1N1) |
| 187 | JF960207 | Spain | 2009 | A/swine/Spain/36/2009 (H1N1) |
| 188 | JQ301942 | Spain | 2009 | A/swine/Spain/36/2009 (H1N1) |
| 189 | JF960208 | Spain | 2009 | A/swine/Spain/37/2009 (H1N1) |
| 190 | JQ301943 | Spain | 2009 | A/swine/Spain/37/2009 (H1N1) |
| 191 | JQ301958 | Spain | 2009 | A/swine/Spain/38/2009 (H1N1) |
| 192 | JQ301962 | Spain | 2009 | A/swine/Spain/38/2009 (H1N1) |
| 193 | JQ301944 | Spain | 2009 | A/swine/Spain/39/2009 (H1) |
| 194 | JQ301957 | Spain | 2009 | A/swine/Spain/40/2009 (H1) |
| 195 | CY067662 | Italy | 2010 | A/swine/Italy/116114/2010 (H1N2) |
| 196 | JF960170 | Spain | 2010 | A/swine/Spain/1/2010 (H1N2) |
| 197 | JF960171 | Spain | 2010 | A/swine/Spain/1/2010 (H1N2) |
